# Significant association between high neutrophil-lymphocyte ratio and poor prognosis in patients with hepatocellular carcinoma: a systematic review and meta-analysis

**DOI:** 10.3389/fimmu.2023.1211399

**Published:** 2023-09-21

**Authors:** Chunhua Xu, Fenfang Wu, Lailing Du, Yeping Dong, Shan Lin

**Affiliations:** ^1^ Shulan International Medical School, Zhejiang Shuren University, Hangzhou, China; ^2^ Department of Central Laboratory, Shenzhen Hospital, Beijing University of Chinese Medicine, Shenzhen, Guangdong, China

**Keywords:** neutrophil-lymphocyte ratio, hepatocellular carcinoma, inflammation, prognostic value, poor prognosis

## Abstract

**Objective:**

Whether neutrophil-lymphocyte ratio (NLR) is an applicative predictor of poor prognosis in patients with hepatocellular carcinoma (HCC) remains controversial. In response to the current conflicting data, this meta-analysis was conducted to gain a comprehensive and systematic understanding of prognostic value of NLR in HCC.

**Methods:**

Several English databases, including PubMed, EMBASE, and the Cochrane Library, with an update date of February 25, 2023, were systematically searched. We set the inclusion criteria to include randomized controlled trial (RCT) studies that reported the prognostic value of serum NLR levels in patients with HCC receiving treatment. Both the combined ratio (OR) and the diagnosis ratio (DOR) were used to assess the prognostic performance of NLR. Additionally, we completed the risk of bias assessment by Cochrane Risk of Bias Assessment Tool.

**Results:**

This meta-analysis ultimately included 16 studies with a total of 4654 patients with HCC. The results showed that high baseline NLR was significantly associated with poor prognosis or recurrence of HCC. The sensitivity of 0.67 (95% confidence interval [CI]. 0.59-0.73); specificity of 0.723 (95% CI: 0.64-0.78) and DOR of 5.0 (95% CI: 4.0-7.0) were pooled estimated from patient-based analyses. Subsequently, the combined positive likelihood ratio (PLR) and negative likelihood ratio (NLHR) were calculated with the results of 2.4 (95% CI: 1.9-3.0) and 0.46 (95% CI: 0.39-0.56), respectively. In addition, area under the curve (AUC) of the summary receiver operating characteristic (SROC) reflecting prognostic accuracy was calculated to be 0.75 (95% CI: 0.71-0.78). The results of subgroup analysis suggested that high NLR was an effective predictive factor of poor prognosis in HCC in mainland China as well as in the northern region.

**Conclusion:**

Our findings suggest that high baseline NLR is an excellent predictor of poor prognosis or relapse in patients with HCC, especially those from high-incidence East Asian populations.

**Systematic review registration:**

https://www.crd.york.ac.uk/prospero/#recordDetails, identifier CRD42023440640.

## Introduction

Hepatocellular carcinoma (HCC) is a malignant tumor that seriously endangers human health, and its pathogenesis is still unclear (1, 2). According to global cancer statistics released in 2022, there were about 906,000 new cases of primary liver cancer and 830,000 deaths worldwide, with China accounting for 42.5% of these cases (3). Unfortunately, 70% to 80% of patients with HCC are in the middle to late stage when diagnosed and have lost the chance of surgical operation (4). Although surgical treatment, chemotherapy, local ablation, molecular targeted therapy and other treatments have been significantly improved, the average survival time of patients with HCC is still very short due to insidious symptoms, rapid development and aggressiveness in early stage (5–7). Therefore, there is an urgent need for sensitive prognostic predictors to help guide the development of treatment plans, improve prognosis, and prolong patient survival.

East Asia is a region with a high prevalence of viral hepatitis B and primary liver cancer (5). Most liver cancers occur after cirrhosis triggered by chronic inflammation, and the interaction between inflammation and tumor is particularly pronounced (8, 9). Systemic inflammation not only plays an important role in tumor development, but also helps to determine the prognosis of patients with HCC (10). The tumor microenvironment of HCC consists mainly of cellular components such as hepatocellular carcinoma cells and inflammatory cells, and non-cellular components such as secreted chemokines and inflammatory factors (11). Some studies have reported that tumor-associated neutrophils can accelerate the proliferation and inhibit the apoptosis of hepatocellular carcinoma cells, promote angiogenesis, and further induce the progression of HCC (12, 13). However, their specific regulation and mechanism of action in HCC are unclear (14). Therefore, whether neutrophil-lymphocyte ratio (NLR), a commonly used clinical index of inflammatory response, can be directly used to discriminate the prognosis of patients with HCC remains to be tested.

More recently, there is growing evidence that increased systemic inflammation in a wide range of cancers is associated with poor cancer-specific survival (15). Both elevated levels of both c-reactive protein (CRP) and NLR can be used to detect the presence of a systemic inflammatory response (16, 17). Although high levels of preoperative serum CRP have been reported to be associated with early recurrence of HCC and poorer survival after hepatectomy, in many hospitals CRP levels are not routinely tested and show non-specific changes after treatment (18). In addition, NLR has an advantage over CRP in terms of inflammatory mechanisms (19). Except for HCC, the expression level of NLRs is closely related to tumor progression, metastasis and prognosis. However, inconsistent data have been generated regarding the predictive power of NLRs for disease progression and overall survival (OS) in HCC (20, 21). Therefore, it is necessary to carry out a meta-analysis to provide a systematic and comprehensive understanding of the predictive value of NLR in HCC.

In this study, we aimed to evaluate the predictive value of high NLR in predicting prognosis and recurrence in patients with HCC. In addition, we considered several sub-analyses to determine the differences in predictive outcomes across countries or regions and dimensional divisions.

## Materials and methods

### Data sources and searches

Several English-language databases were systematically searched, including PubMed, EMBASE and the Cochrane Library, from creation to 25 February 2023. The following search terms were used (**Supplementary material 1**): (“hepatocellular carcinoma” [Mesh], or “liver cancer” [Mesh], or “hepatoma” [Mesh], or “hepatic carcinoma” [Mesh]), and (“inflammatory markers” [Title/Abstract], OR “neutrophil-to-lymphocyte ratio” [Title/Abstract], OR “neutrophil lymphocyte ratio” [Title/Abstract], OR “neutrophil to lymphocyte ratio” [Title/Abstract]). The summary with full results section was included in the present study. The bibliographies of the retrieved articles were checked manually for additional references. This meta-analysis was conducted based on PRISMA statements (Preferred Reporting Items for Systematic Reviews and Meta-Analyses) (22, 23). This present meta-analysis has been submitted to PROSPERO (ID 440640).

### Study selection

All citations are reviewed in order. Search for full text of potentially relevant articles by title or abstract, and two investigators (Chunhua Xu and Shan Lin) independently reviewed to identify eligible studies. Disagreements about eligibility were resolved through discussions with the arbitrator (Fenfang Wu). Studies that explicitly met the following inclusion criteria were considered for inclusion: (1) serum NLR levels were measured prior to formal treatment; (2) participants in human studies were ≥18 years old; (3) sample size was >20; (4) randomized controlled trials (RCTs) are observational; and (5) false positive (FP), sufficient true positive (TP), false negative (FN), as well as true negative (TN) data were provided to calculate the predictive power of NLR in patients with HCC. Studies meeting the following exclusion criteria were excluded. (1) lack of information on the prognostic accuracy of the control or experimental groups; (2) animal or *in vitro* studies only; (3) presence of duplicate data or insufficient information; and (4) article type of review, poster, commentary, editorial or supplemental question.

### Extraction of data

The data of each experiment were collected by Xu Chunhua and Lin Shan respectively. All disagreements or differences among the reviewers were discussed and evaluated by a third-party reviewer (Lailing Du) until a consensus was reached. Pre-specified data for each study included a request to record and recalculate the following variables: first author, country or region, publication year, study design, entry time, sample size (male), median age (years), area under the curve (AUC; 95% confidence interval [CI]), sensitivity, specificity, and baseline NLR cutoff value.

### Quality assessment

In this study, we applied the Cochrane Risk of Bias Assessment Tool with 2 independent reviewers (Chunhua Xu and Shan Lin) to assess the quality of the articles (24). The tool includes six domains: allocation concealment, random sequence generation, blinding, incomplete outcome data, outcome reporting options and other bias resources. All six domains were assessed as “risk of bias” and “applicability issues”, and each item was judged as “yes”, “no” or “unclear”.

Additionally, eligible studies were assessed using the Newcastle-Ottawa scale (NOS) (25). Estimates of study quality were based on comparability, selection and exposure by a star system of up to 9 stars. The quality of each trial was defined as 0-3 stars as poor, 4-6 stars as fair and 7-9 stars as good. Finally, the quality assessment of the NOS was based on previous studies with some modifications (26).

### Statistical analysis

In this study, we used Stata version 12.0 (StataCorp, College Station, TX, USA) software for all statistical analyses of TN, TP, FP and FN rates for each study, as well as diagnostic odd ratio (DOR) sensitivity, specificity, positive likelihood ratio (PLR), as well as negative likelihood ratios (NLHR) were fully assessed. *P*-values <0.05 for the Q statistic as well as *I* ^2^ values >50% for the *I* ^2^ statistic were all considered to be statistically significant heterogeneity (27). When heterogeneity was high (*I* ^2^ > 50%), a random effects model was applied (28). Hardy-Weinberg equilibrium (HWE) of each study in the control group was considered statistically significant by Pearson’s χ2 test with *p-* value <0.05 (29).

In addition, to evaluate the predictive performance of NLR in patients with HCC, we performed pooled receiver characteristics (SROC) curves and pooled sensitivity and specificity forest plots by assessing AUC as a summary metric (30). Then, subgroup analyses were also carried out on a geographic or regional basis. Finally, we detected possible publication bias using Begg’s and Egger’s tests, and considered statistically significant with a *p*-value < 0.05 (31, 32).

## Results

### Literature search

**Figure 1:**
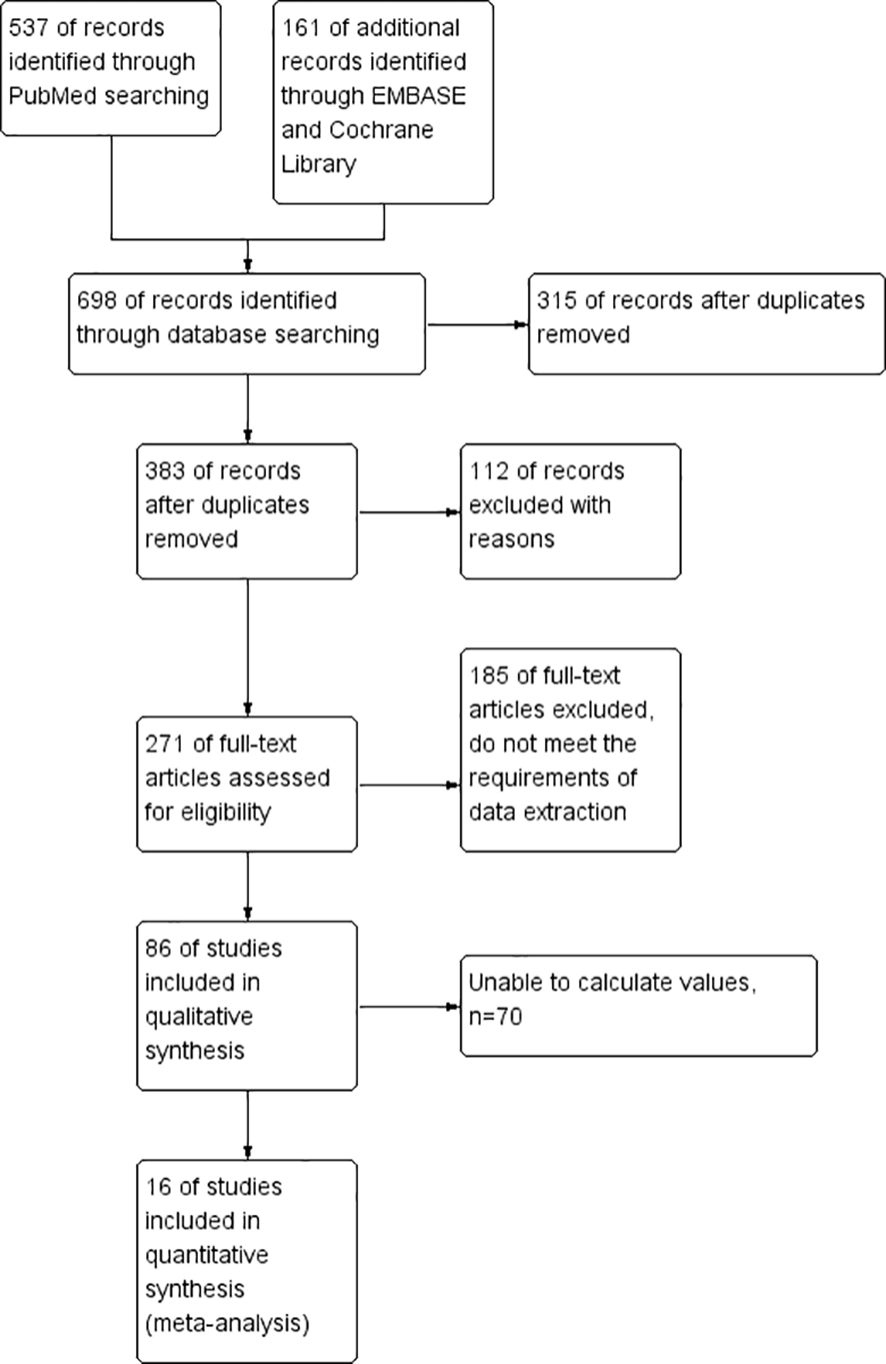
The process of selecting studies for inclusion in this meta-analysis.

Initially, a search of an electronic database yielded 698 potentially relevant papers, but after culling the duplicates, 315 papers were eliminated. From the title and abstract, 112 studies were obviously irrelevant and were therefore excluded. After the remaining 271 papers were reviewed, 185 papers were rejected and 70 papers were rejected. In the end, 16 papers were selected. Through 16 literatures, including 4654 cases, the role of NLR in the prognosis of HCC was evaluated by using the Meta method. **Figure 1** details the step-by-step screening procedure for the included trials.

### The characteristics and quality of the included studies

All 16 papers are written in English. To assess the quality of the included trials, basic data were extracted, as shown in **Table 1**. Of the 16 trials, 15 were in East Asia and the remaining 1 was in West Africa (43), including 11 in mainland China (34, 37, 39–41, 44, 46–50), 1 in South Korea (38), 2 in Taiwan (33, 51), and 1 in Japan (36). All studies are from single-center clinical trials between 2012 and 2022. All the 16 observational studies included 4654 HCC patients, including 3986 in mainland China, 213 in Korea, 40 in Japan, 311 in Taiwan, and 104 in mainland China. **Table 2** summarizes the predictive power of NLR in the prognosis of patients with HCC. The AUC is between 0.602 and 0.855, and the threshold is between 1.505 and 3.290. In addition, sensitivities and specificities were calculated or given for the included tests in the ranges 0.301 to 0.840 and 0.440 to 0.887, respectively.

**Table 1:** Main characteristics of the enrolled studies.

Author (Year of publication)	Regions (City)	Sample size (male)	Enrollment period	Median age	NOS
Chen et al. (2012) (33)	Taiwan (Taipei)	158 (95)	2003.07-2010.12	65.7 (31.8-82.8)	8
Dai et al. (2019) (34)	China (Guangzhou)	195 (174)	2005.03-2013.05	51(42–59)	8
Du et al. (2019) (35)	China (Xi’an)	230 (174)	2000.01-2012.12	44(20–66)	7
Eso et al. (2021) (36)	Japan (Kyoto)	40 (35)	2020.10-2021.08	70.5(53–82)	7
Gao et al. (2015) (37)	China (Beijing)	825 (690)	2008.10-2012.05	54.5(25–75)	8
Hu et al. (2016) (38)	Korea (Suwon)	213 (166)	2001.03-2011.12	53(20–79)	8
Li et al. (2014) (39)	China (Beijing)	506 (420)	2005.04-2014.04	59.2(28–85)	8
Liu et al. (2016) (40)	China (Nanjing)	223 (189)	2004.07-2011.04	54(21–82)	7
Liu et al. (2017) (41)	China (Chengdu)	760 (643)	2007.01-2013.12	56.5(19–89)	7
Lo et al. (2021) (42)	Taiwan (Taipei)	153 (114)	2007.12-2018.08	64(56–74)	8
Mahassadi et al. (2021) (43)	Cote d’Ivoire (Abidjan)	104 (61)	2012.01-2015.12	49.5(24–86)	7
Qu et al. (2022) (44)	China (Changzhou)	215 (178)	2010.01-2018.08	59.1	7
Sun et al. (2019) (45)	China (Beijing)	47 (44)	2008-2017	40(30–44)	7
Tan et al. (2018) (46)	China (Qingdao)	402 (299)	2008.09-2017.05	51.7(18–92)	8
Wang et al. (2019) (14)	China (Changsha)	239 (200)	2012-2015	50.14(38–62)	8
Wu et al. (2018) (47)	China (Beijing)	344 (292)	2010.05-2014.04	54 (19–84)	8

NR, no result; NOS, Newcastle-Ottawa Scale.

**Table 2:** The predictive value of NLR for poor prognosis of hepatocellular carcinoma.

Study	AUC	95% CI	Cut-off value(ng/mL)	Sensitivity(%)	Specificity(%)	Number of patients			
						TP	FP	FN	TN
Chen et al. (2012) (33)	0.630	0.520-0.720	2.400	0.730	0.470	59	41	22	36
Dai et al. (2019) (34)	0.650	0.545-0.755	2.000	0.750	0.440	52	71	17	55
Du et al. (2019) (35)	0.625	0.527-0.732	2.270	0.639	0.653	57	49	32	92
Eso et al. (2021) (36)	0.746	No result	3.210	0.808	0.769	12	6	3	18
Gao et al. (2015) (37)	0.811	No result	2.700	0.662	0.848	220	75	112	418
Hu et al. (2016) (38)	0.643	No result	1.505	0.775	0.486	93	48	27	45
Li et al. (2014) (39)	0.824	No result	2.140	0.780	0.690	143	100	40	223
Liu et al. (2016) (40)	0.606	No result	2.750	0.301	0.887	16	19	37	151
Liu et al. (2017) (41)	0.664	0.630-0.698	2.200	0.752	0.545	393	108	129	130
Lo et al. (2021) (42)	0.762	0.682-0.841	2.400	0.628	0.844	57	10	33	53
Mahassadi et al. (2021) (43)	0.680	No result	2.500	0.530	0.750	21	16	18	49
Qu et al. (2022) (44)	0.602	No result	3.290	0.595	0.730	49	36	33	97
Sun et al. (2019) (45)	0.681	No result	3.090	0.670	0.730	14	7	7	19
Tan et al. (2018) (46)	0.855	No result	2.200	0.840	0.860	210	21	40	131
Wang et al. (2019) (50)	0.630	0.560-0.710	2.92	0.510	0.780	53	30	51	105
Wu et al. (2018) (47)	0.634	No result	2.15	0.488	0.769	55	54	57	178

### Assessment of methodological quality and publication bias

**Figure 2:**
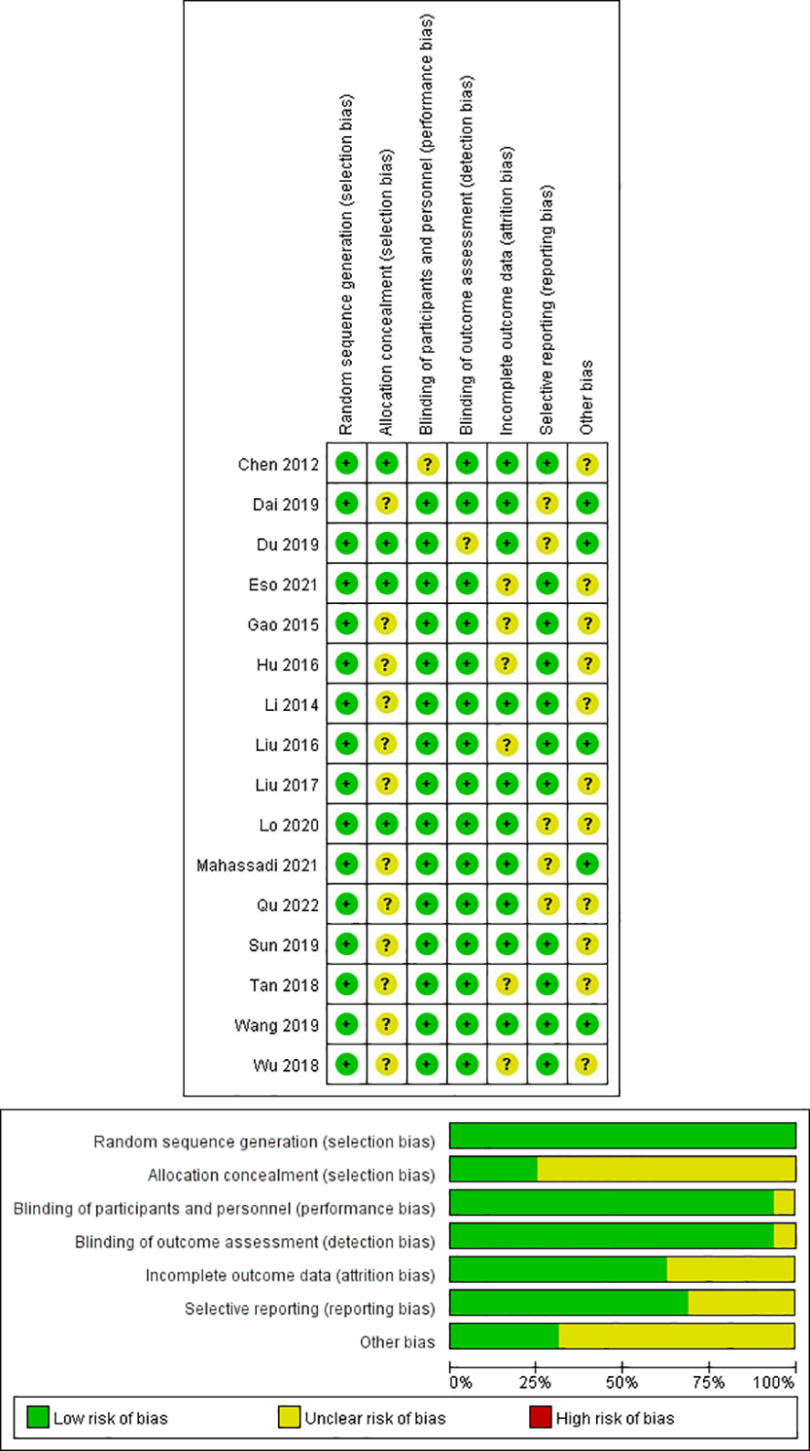
Quality assessment of included eligible studies by the Cochrane Risk of Bias Assessment Tool.

In this study, all trials had detailed inclusion criteria and excluded patients. Second, the quality of each included study (all with a Cochrane score of 10 or above) was assessed using the Cochrane risk of bias tool (Cochrane). In addition, the overall quality of the included studies was average. **Figure 2** shows the results of the Cochrane evaluation.

**Figure 3:**
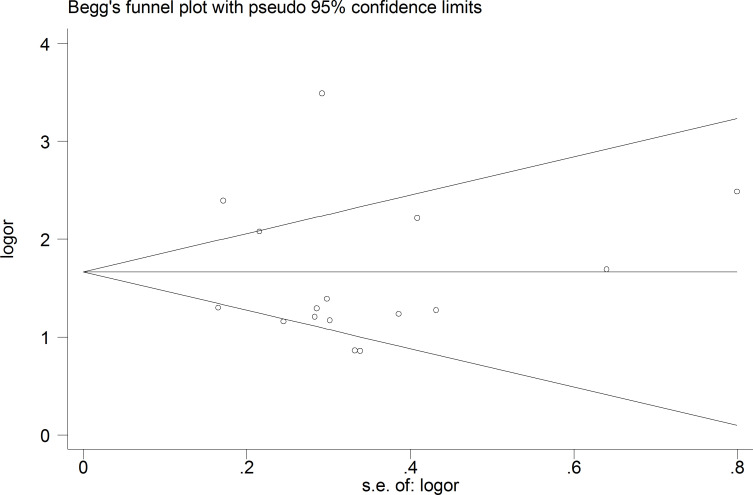
The Begg’s funnel plot for testing publication bias.

**Figure 4:**
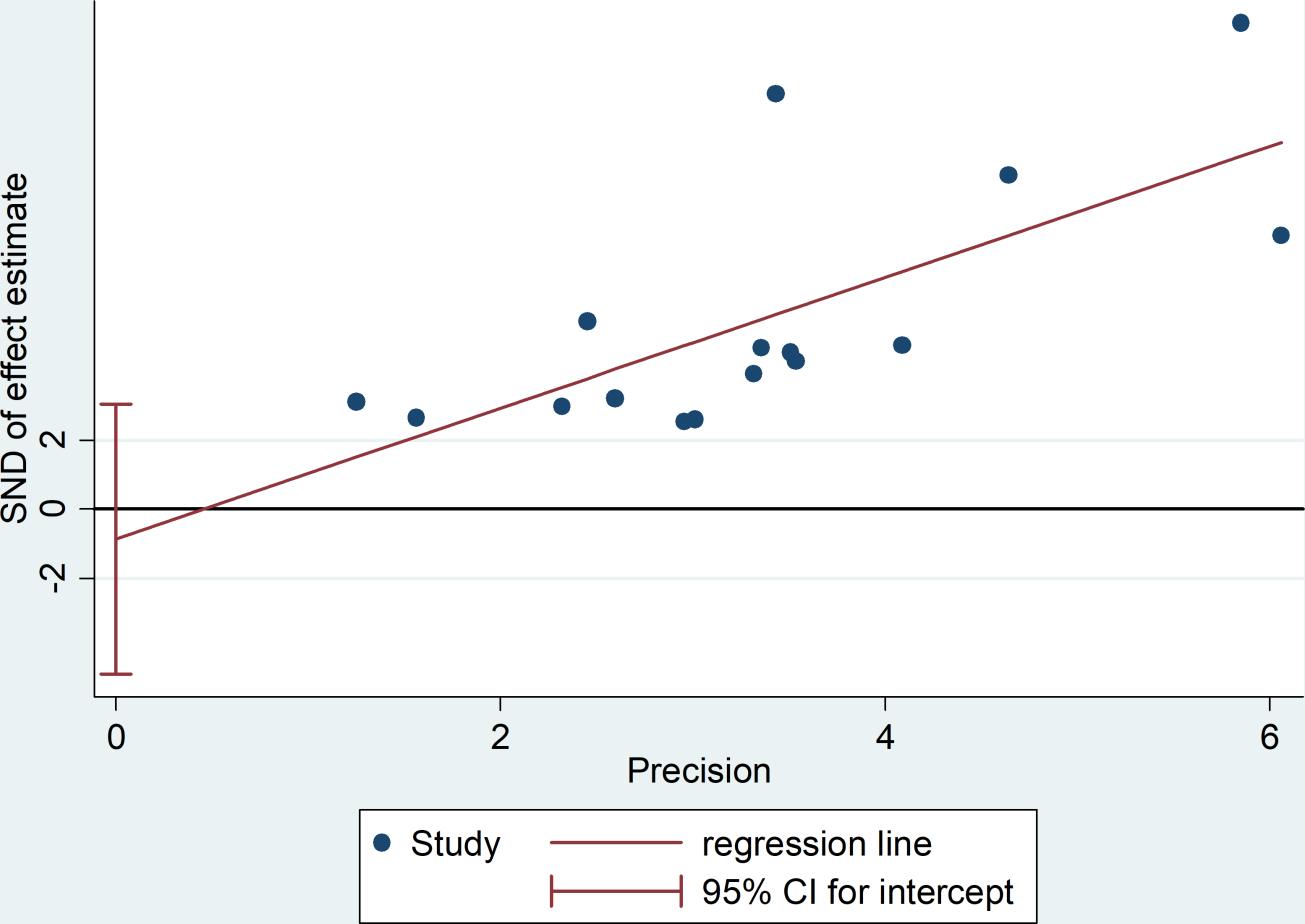
The Egger’s funnel plot for testing publication bias.

Moreover, the probability of publication bias was evaluated by Begg’s and Egger’s funnel plot, and the results were shown in **Figures 3** and **4**, respectively. It was suggested that there appears to be publication bias in the present study. Even though the overall risk of bias seemed low, the results do not appear to be significantly changed by studies that have not yet been published.

### NLR for predicting prognosis in patients with HCC

**Figure 5:**
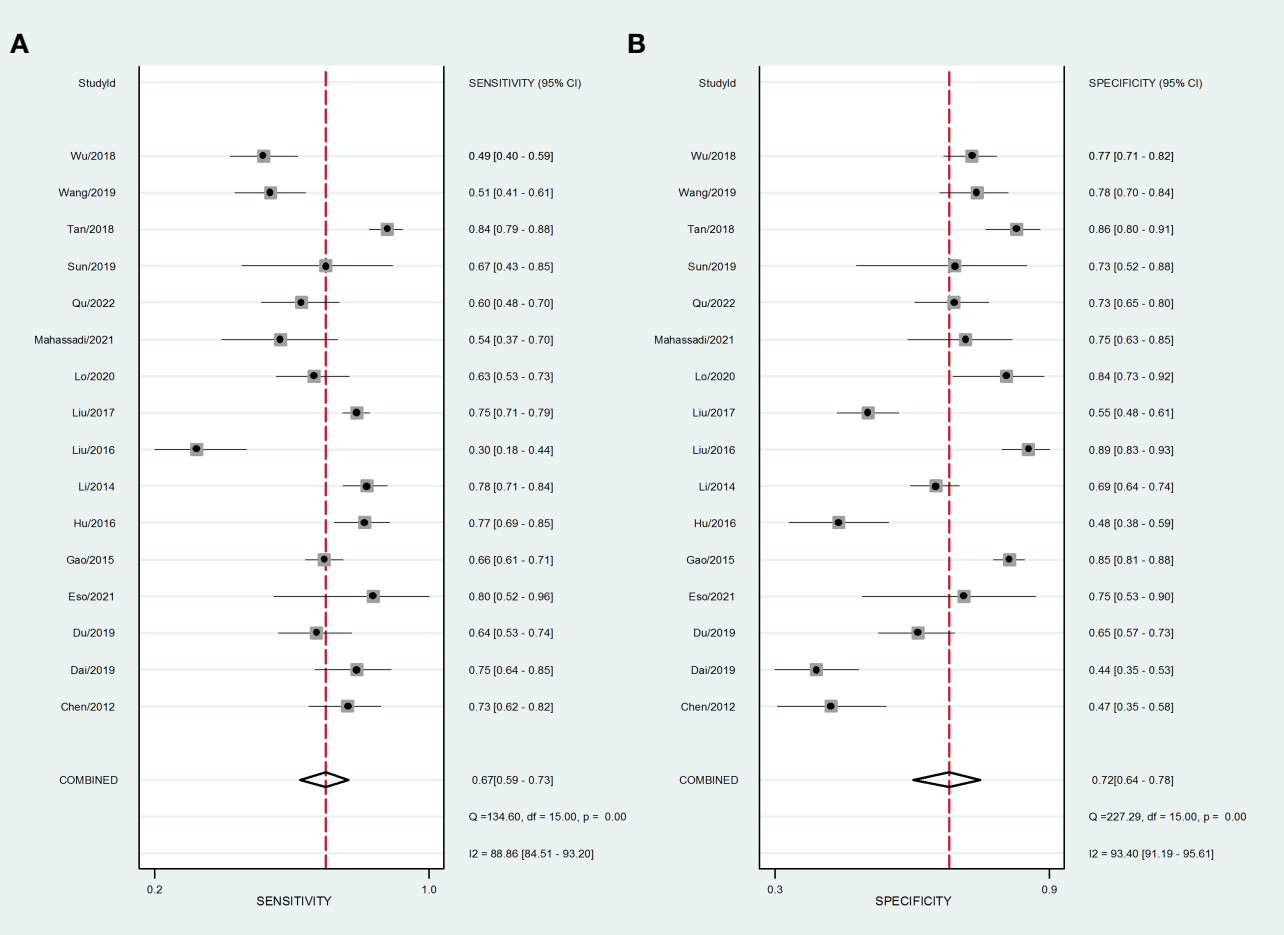
Forest plot of sensitivity and specificity of high NLR predicting HCC prognosis. **(A)** Sensitivity. **(B)** Specificity.

Sixteen data sets were extracted from 16 qualified literatures (**Table 2**), including AUC, 95% CI, optimal cut-off value of NLR, sensitivity, specificity, and TP, FP, FN, TN, etc. 16 studies examined the predictive value of NLR as a biomarker of prognosis in HCC patients with a total population of 4654. **Table 2** summarizes the combined data from these trials. For the evaluation of the efficacy of NLR, it had a pooled sensitivity of 0.68 (95% CI: 0.58-0.77) (**Figure 5A**), specificity of 0.73 (95% CI: 0.61-0.82) (**Figure 5B**), and PLR of 2.5 (95% CI: 1.8-3.6), as well as NLHR of 0.43 (95% CI: 0.33-0.57). Subsequently, it had a pooled DOR of 6.347 (95% CI: 5.450-7.391) according to a random effects model.

**Figure 6:**
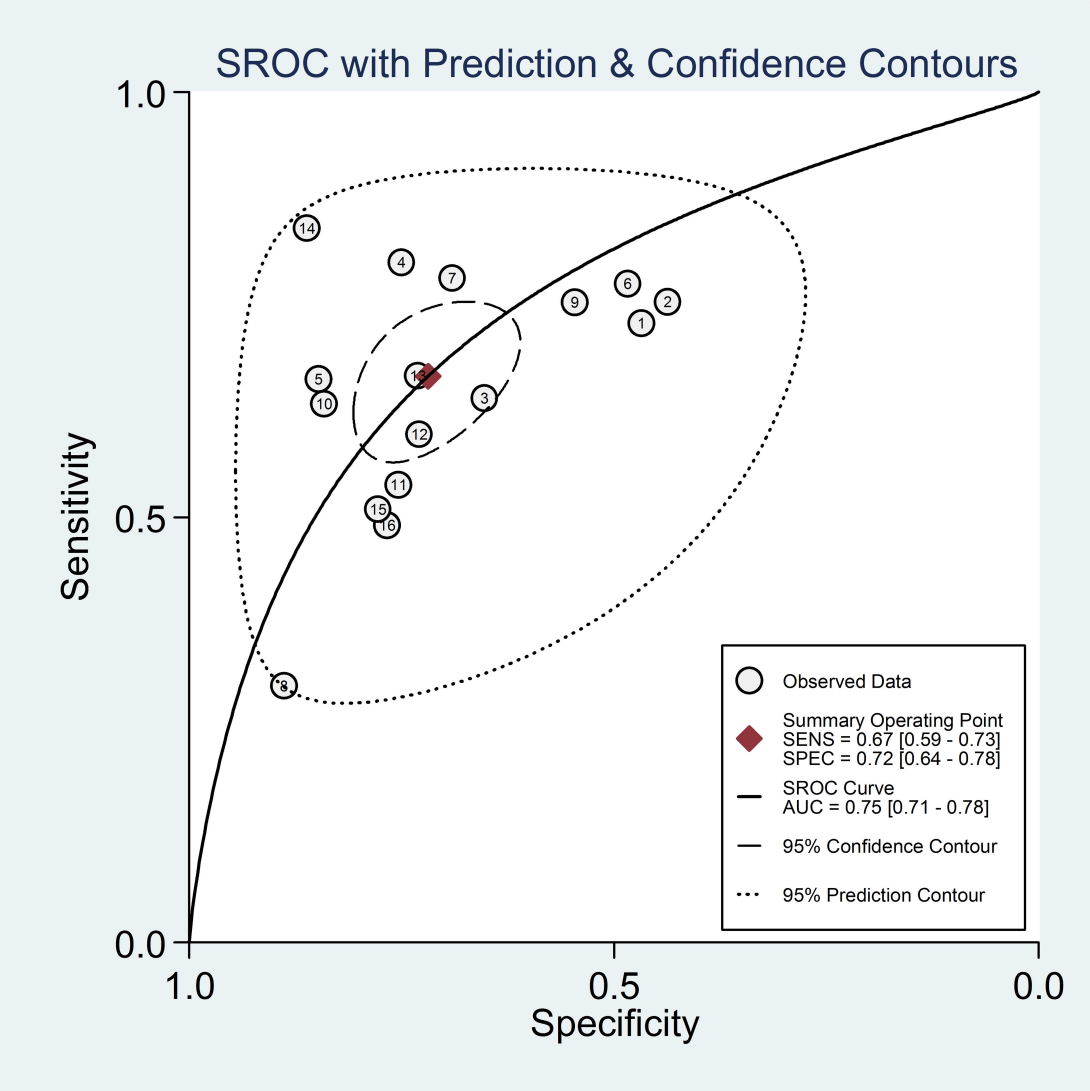
Forest plot of AUC of high NLR predicting HCC prognosis.

**Figure 7:**
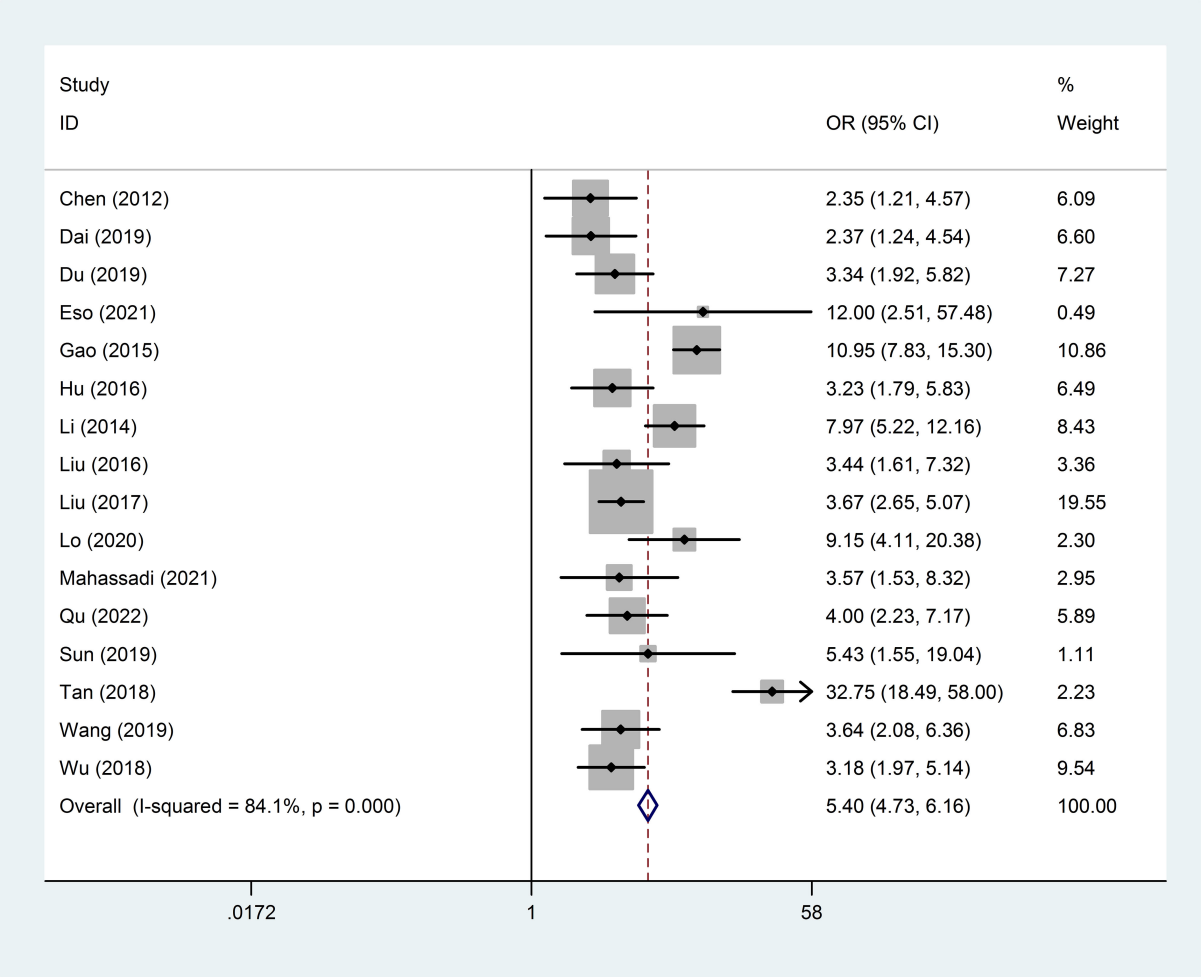
Forest plot of the predictive value of high NLR on the prognosis of HCC patients.

In addition, forest plot of AUC of high NLR predicting HCC prognosis was carried out and shown in **Figure 6**. It was suggested that no “shoulder arm” pattern was seen in the SROC space, indicating the absence of a threshold effect. Furthermore, the AUC for the prognostic accuracy of SROC prediction was calculated as 0.76 (95% CI: 0.72-0.80). Moreover, the predictive value of NLR for prognosis of patients with HCC is summarized in **Figure 7**. Therefore, it is not difficult to conclude from the results that high baseline NLR is significantly associated with poor prognosis of HCC.

### Subgroup analysis

To further understand the differences in the predictive value of high NLR for recurrence and poor prognosis of HCC in populations from different countries (or regions) and in populations with different dimensional gradations, we performed a systematic subgroup analysis, and the results were shown in **Table 3**. There were significant differences in the prognostic value of NLR in patients with HCC according to the comparison of DOR and AUC. In the subgroup regional analysis, the DOR and AUC of NLRs in mainland China were higher than those in Korea (DOR, 5; AUC, 0.75 vs. DOR, 3; AUC, 0.64), and Côte d’Ivoire (DOR, 5; AUC, 0.75 vs. DOR, 4; AUC, 0.68), but significantly lower than those in Japan (DOR, 5; AUC, 0.75 vs. DOR, 12; AUC, 0.75), suggesting that the prognostic value of high NLR in mainland Chinese patients with HCC for HCC recurrence and poor prognosis value was superior to that of patients with HCC in Korea and Côte d’Ivoire, but inferior to Japan. In the subgroup latitude analysis, the prognostic value of high NLR for HCC recurrence and poor prognosis was better in patients with HCC at high latitudes than in patients with HCC at low latitudes (DOR, 7; AUC, 0.79 vs. DOR, 4; AUC, 0.71). Interestingly, combining the results of the above subgroup analyses, the prognostic predictive value for NLR was quite high in the high latitudes of East Asia, which may be related to the high incidence of HCC in this region.

**Table 3:** Subgroup analysis for regions and latitude distribution.

Studies	Number		Sensitivity	Specificity	PLR	NLHR	DOR	AUC
Regions	11	China	0.65(0.56-0.74)	0.74 (0.65-0.81)	2.5 (1.9-3.3)	0.47(0.37-0.60)	5(3–8)	0.75 (0.71-0.79)
	1	Korea	0.775	0.486	No result	No result	3(2–6)	0.64
	2	Taiwan	No result	No result	No result	No result	5(3–7)	No result
	1	Japan	0.81	0.77	No result	No result	12(3–57)	0.75
	1	Cote d’Ivoire	0.53	0.75	No result	No result	4(2–8)	0.68
Cutoff identification	12	ROC	0.68(0.63-0.73)	0.70(0.62-0.77)	2.3(1.8-2.8)	0.45(0.40-0.52)	5(4–7)	0.74(0.70-0.78)
Age	10	Median ag ≤55	0.63(0.53-0.73)	0.74(0.64-0.82)	2.5(1.8-3.4)	0.49(0.38-0.64)	5(3–8)	0.74(0.70-0.78)
	6	Median age >55	0.71(0.66-0.76)	0.68(0.57-0.77)	2.2(1.7-2.9)	0.42(0.36-0.50)	5(4–8)	0.75(0.71-0.79)
Gender	10	Male ≤200	0.64(0.56-0.72)	0.68(0.57-0.77)	2.0(1.6-2.5)	0.53(0.46-0.60)	4(3–5)	0.70(0.66-0.74)
	6	Male >200	0.69(0.58-0.78)	0.76(0.67-0.83)	2.9(2.0-4.2)	0.41(0.29-0.58)	7(4–13)	0.79(0.75-0.82)
Latitude distribution	8	High-latitude	0.72(0.63-0.79)	0.74 (0.65-0.81)	2.7 (2.0-3.8)	0.39(0.29-0.52)	7(4–12)	0.79 (0.75-0.82)
	8	Low-latitude	0.62 (0.51-0.72)	0.70 (0.58-0.80)	2.1 (1.6-2.6)	0.54 (0.47-0.63)	4(3–5)	0.71 (0.66-0.74)

Generally, the optimal cut-off value of NLR was determined by using receiver operating characteristic (ROC) curves. For the subgroup analysis of method of NLR cutoff identification (**Table 3**), the method of ROC showed DOR and AUC of 5(4–7) and 0.74(0.70-0.78), respectively. For the subgroup analysis of age, the group of median age ≤55 showed similar DOR and AUC to the group of median age >55 (DOR, 5(3–8); AUC, 0.74(0.70-0.78) vs. DOR, 5(4-8); AUC, 0.75(0.71-0.79)). Additionally, for the subgroup analysis of gender, the group of male >200 showed significantly higher DOR and AUC compared with the group of male ≤200 (DOR, 7(4-13); AUC, 0.79(0.75-0.82) vs. DOR, 4(3-5); AUC, 0.70(0.66-0.74)).

### Analysis of sensitivity and heterogeneity

Each study included in the meta-analysis was removed each time to investigate the impact of a single data set on the combined OR. The results of the sensitivity analysis demonstrate the robustness of the findings in this study (data not shown).

In addition, possible explanations for heterogeneity were evaluated by meta-regression and subgroup analyses. The factor of high-latitude (*I* ^2 =^ 88.2%, *P* = 0.000) and mainland China (*I* ^2 =^ 87.8%, *P* ≤ 0.001) may be the main source of heterogeneity, while the factor of low-latitude (*I* ^2 =^ 18.0%, *P* = 0.287) may not be a source of heterogeneity in the predictive value of NLR. For subgroup analysis with heterogeneity, the random effects model was used to combine the effect size. For subgroup analyses without heterogeneity, fixed-effect models were used to combine effect sizes.

## Discussion

Factors affecting the prognosis of patients with primary HCC include tumor-related factors (such as the volume and load of the tumor) and patient-related factors (52). In addition, the inflammatory response plays an important role in the development of tumorigenesis, not only participating in the proliferation and metastasis of tumor cells, but also promoting the immune escape of tumors and affecting the responsiveness to treatment (53, 54). Here, in this meta-analysis of 16 studies comprising 4654 patients with HCC, the results showed that high baseline NLR was significantly associated with poor prognosis or recurrence of HCC, which suggested that NLR is an excellent predictor of poor prognosis or relapse in patients with HCC. In subgroups stratified by regions and latitude distribution, high NLR had better predictive value for poor prognosis of HCC in mainland China as well as in northern regions.

Systemic inflammatory responses are associated with the recurrence of certain tumors (55). The interaction of different types of immune cells leads to tumor immune escape and subsequently promotes tumor progression (56). Among the systemic inflammatory indicators, NLR and PLR are hot spots in the prognostic studies of various tumors (57). Neutrophils are involved in the progression of cancer in multiple stages and aspects (58). On the one hand, neutrophils directly promote tumor growth by secreting chemokines and cytokines and actively recruiting other tumor-supporting cells (59). On the other hand, tumor-associated neutrophils are involved in mediating the angiogenic switch and promoting tumor angiogenesis (60). At the same time, enzymes that degrade and modify the extracellular matrix are secreted to promote tumor cell invasion (61). In addition, lymphocytes reflect the body’s anti-tumor immunity, and changes in the ratio of the two are associated with an imbalance between the two types of cells, which reflects a disruption of the dynamic balance between the immune state and tumor inflammation (62).

In recent years, some simple indicators of inflammation have been used to predict tumor recurrence and metastasis with good practical value (63). NLR can be used to evaluate systemic inflammatory changes and can reflect the possible balance between preneutrophil neoplastic inflammation and lymphocyte-dependent antitumor immunity (64). Unfortunately, the specific mechanisms underlying the relationship between NLR and tumor recurrence and survival of tumor patients are not well understood, but the results of some basic studies may partially explain the mechanisms (65). One plausible explanation is that elevated neutrophils lead to the production of more inflammatory mediators, which in turn affect the tumor microenvironment and promote tumor recurrence and metastasis (66). Similarly, the role of neutrophils in tumourigenesis is to secrete high levels of vascular endothelial growth factor, IL-1 and IL-6, which in turn promote the production of tumor blood vessels, leading to tumor growth, development and metastasis (67). An increase in NLR indicates a relative increase in neutrophils or a relative decrease in lymphocytes, leading to a shift in the dynamic balance of inflammation towards tumor promotion. As suggested by the results of the present study, an increase in the NLR ratio favors the inflammatory response of the tumor, suggesting a propensity for malignancy to develop, proliferate and metastasize. Conversely, a weakening of the index reflects an increased antitumor function.

Recently, several studies showed that the NLR may be correlated with the prognosis of patients with HCC, but most of them were initially treated by liver transplantation (LT) (21, 45, 68). The findings obtained by Xiao et al. on 3094 patients showed that high NLR was associated with poor overall survival (OS) and disease free survival (DFS) in HCC initially treated by LT (21). Furthermore, Xu et al. conducted a study on 1936 patients and showed that elevated pretransplant NLR may be used as a new prognostic predictor after LT for HCC (68). Additionally, Sun et al. conducted a meta-analysis on 1687 patients and suggested that elevated preoperative NLR is associated with poor prognosis in HCC patients treated with LT (45). In the present study, the scope of the study was not limited to LT, which not only had a larger clinical sample size with 4654 patients, but also reflected the correlation between NLR and HCC prognosis more comprehensively.

Intriguingly, it is worth mentioning that there were other biomarkers not only inflammatory ones, such as APRI and CRP. It is reported that high APRI levels are associated with poor OS and DFS in the patients with HCC, and pretreatment APRI can be used as an independent prognostic factor, but it is necessary to incorporate other predictive prognostic systems to ensure accuracy (69). Furthermore, the significance of CRP has been demonstrated as a predictor of survival in HCC, but the current opinion on the prognostic role of CRP in HCC is still controversial (70). Therefore, even though it has been reported that APRI and CRP might be candidates as prognostic biomarkers in HCC, the clinical value of them in HCC were still inconsistent and debatable for many reasons, such as limited sample sizes. In this present study, an adequate sample size was included to evaluate the predictive value of high NLR in predicting prognosis and recurrence in patients with HCC, which could gain a comprehensive understanding of prognostic value of NLR in HCC.

There are some limitations in this study. First, the included studies were retrospective, and patients had different late treatment regimens, which may have had an impact on patient prognosis. Second, the levels of preoperative neutrophil and lymphocyte counts are susceptible to a variety of factors, such as hepatitis and the degree of cirrhosis. Furthermore, it was shown that there is low level of publication bias. Even though the overall risk of bias may be low, it can be avoided by including more published articles to inflate the sample size in the future. In addition, most of the original studies demonstrated an association between high baseline NLR and poor prognosis of HCC, which may be due to the ease of publication of positive results, ultimately making it difficult to find more controversial studies. Finally, the cutoff value for defining high NLR differed in studies, and it is essential that they be unified before high NLR can be utilized in clinical prognostication for HCC.

## Conclusion

A high level of NLR is an excellent prognostic indicator for HCC and can be used to predict early relapse, late relapse and long-term survival of HCC. It can be an effective reference indicator for clinicians to judge the prognosis of patients and adjust the treatment in a timely manner. Even so, larger and better-designed investigations are needed to further fully elucidate the predictive value of NLR for the prognosis of HCC patients.

## Data availability statement

The original contributions presented in the study are included in the article/**Supplementary Material**. Further inquiries can be directed to the corresponding author.

## Author contributions

CX and SL contributed to the conception and design. CX and FW contributed to the administrative support. CX and LD contributed to the provision of study materials or patients. SL and YD contributed to the collection and assembly of data. CX contributed to the data analysis and interpretation. All authors agree to the submission for publication.
