# Supplementary material for: Significant association between high neutrophil-lymphocyte ratio and poor prognosis in patients with hepatocellular carcinoma: a systematic review and meta-analysis
Source: Front Immunol. 2023 Sep 21;14:1211399. doi: 10.3389/fimmu.2023.1211399 (PMC10551132; doi:10.3389/fimmu.2023.1211399)
Supplement: Supplementary file 1 [file DataSheet_1.docx]

Search strategy for PubMed

| Search number | Query | Search Details |
| --- | --- | --- |
| 3 | (#1) AND (#2) | ("carcinoma, hepatocellular"[MeSH Terms] OR "liver neoplasms"[MeSH Terms] OR "carcinoma, hepatocellular"[MeSH Terms] OR "carcinoma, hepatocellular"[MeSH Terms]) AND ("inflammatory markers"[Title/Abstract] OR "neutrophil to lymphocyte ratio"[Title/Abstract] OR "neutrophil lymphocyte ratio"[Title/Abstract] OR "neutrophil to lymphocyte ratio"[Title/Abstract]) |
| 2 | (((inflammatory markers[Title/Abstract]) OR (neutrophil-to-lymphocyte ratio[Title/Abstract])) OR (neutrophil lymphocyte ratio[Title/Abstract])) OR (neutrophil to lymphocyte ratio[Title/Abstract]) | "inflammatory markers"[Title/Abstract] OR "neutrophil to lymphocyte ratio"[Title/Abstract] OR "neutrophil lymphocyte ratio"[Title/Abstract] OR "neutrophil to lymphocyte ratio"[Title/Abstract] |
| 1 | (((hepatocellular carcinoma[MeSH Terms]) OR (liver cancer[MeSH Terms])) OR (hepatoma[MeSH Terms])) OR (hepatic carcinoma[MeSH Terms]) | "carcinoma, hepatocellular"[MeSH Terms] OR "liver neoplasms"[MeSH Terms] OR "carcinoma, hepatocellular"[MeSH Terms] OR "carcinoma, hepatocellular"[MeSH Terms] |

Search strategy for EMBASE

| No. | Query Results |
| --- | --- |
| #3. | #1 AND #2 |
| #2. | 'inflammatory markers':ab,ti OR 'neutrophil-to-lymphocyte ratio':ab,ti OR 'neutrophil lymphocyte ratio':ab,ti OR 'neutrophil to lymphocyte ratio':ab,ti |
| #1. | 'hepatocellular carcinoma':ab,ti OR 'liver cancer':ab,ti OR hepatoma:ab,ti OR 'hepatic carcinoma':ab,ti |

Search strategy for Cochrane Library

| ID | Search Hits |
| --- | --- |
| #1 | (hepatocellular carcinoma):ti,ab,kw OR (liver cancer):ti,ab,kw OR (hepatoma):ti,ab,kw OR (hepatic carcinoma):ti,ab,kw (Word variations have been searched) |
| #2 | (inflammatory markers):ti,ab,kw OR (neutrophil-to-lymphocyte ratio):ti,ab,kw OR (neutrophil lymphocyte ratio):ti,ab,kw OR (neutrophil to lymphocyte ratio):ti,ab,kw (Word variations have been searched) |
| #3 | #1 AND #2 |
